# Complex Permittivity of Planar Building Materials Measured With an Ultra-Wideband Free-Field Antenna Measurement System

**DOI:** 10.6028/jres.112.005

**Published:** 2007-02-01

**Authors:** Ben Davis, Chriss Grosvenor, Robert Johnk, David Novotny, James Baker-Jarvis, Michael Janezic

**Affiliations:** National Institute of Standards and Technology, Boulder, CO 80305-3328

**Keywords:** digital signal processing, free-field measurement system, genetic algorithm, material properties, TEM half-horn antennas, transmission, ultra-wide-band

## Abstract

Building materials are often incorporated into complex, multilayer macrostructures that are simply not amenable to measurements using coax or waveguide sample holders. In response to this, we developed an ultra-wideband (UWB) free-field measurement system. This measurement system uses a ground-plane-based system and two TEM half-horn antennas to transmit and receive the RF signal. The material samples are placed between the antennas, and reflection and transmission measurements made. Digital signal processing techniques are then applied to minimize environmental and systematic effects. The processed data are compared to a plane-wave model to extract the material properties with optimization software based on genetic algorithms.

## 1. Introduction

The NIST Time-Domain Fields Project has developed a nondestructive, ultra-wideband (UWB) measurement system to measure complex permittivities of building materials. The purpose is to develop a database of material permittivities for use by engineers for modeling wireless signal propagation in buildings and related applications.

The NIST system consists of an aluminum ground plane, two TEM horn antennas, precision microwave cables, and a vector network analyzer. A wall of sample building material is placed between the antennas, and both transmission and reflectivity are measured over a wide frequency range. The stepped-frequency S-parameters are then post-processed to isolate the sample response and reduce systematic effects due to the environment. The net result is a set of gated S-parameters. Material properties are then extracted by an optimization process based on plane-wave assumptions and the gated S-parameters. This optimization process utilizes a genetic algorithm (GA) [1] that provides accurate estimates of the electrical properties of a sample under test. This system has the marked advantage over previous systems in that it permits the measurement of very heavy wall samples without the need of a sample holder. The system also has high bandwidth, range (spatial) resolution, and measurement fidelity.

This paper consists of six sections: Section 2 describes the measurement system. Section 3 introduces the measurement sequence and the initial data processing. Section 4 describes the mathematical model and how the GA is used to optimize material parameter estimates for two candidate materials, cross-linked polystyrene and concrete. Section 5 compares the results obtained thus far with other measurement techniques and published results. Section 6 presents conclusions.

## 2. The Measurement System

**Fig. 1:**
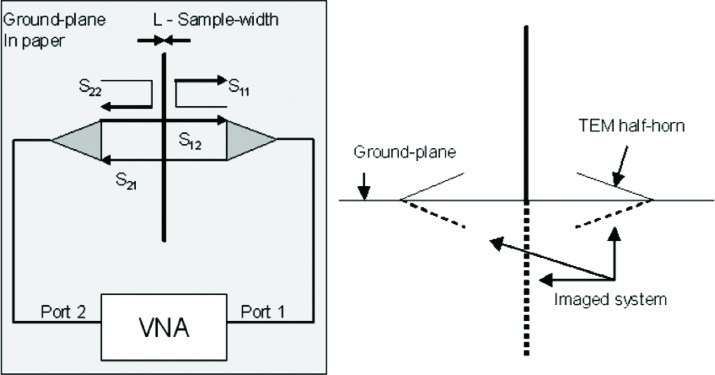
Plan view and profile view of sample under measurement.

**Fig. 2:**
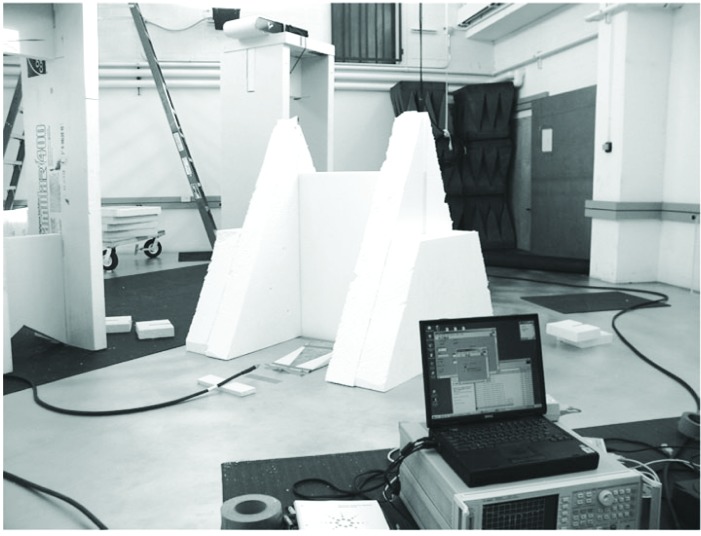
1.2 m × 1.2 m cross-linked polystyrene in styrofoam holders for measurement on a laboratory ground-plane.

**Fig. 3:**
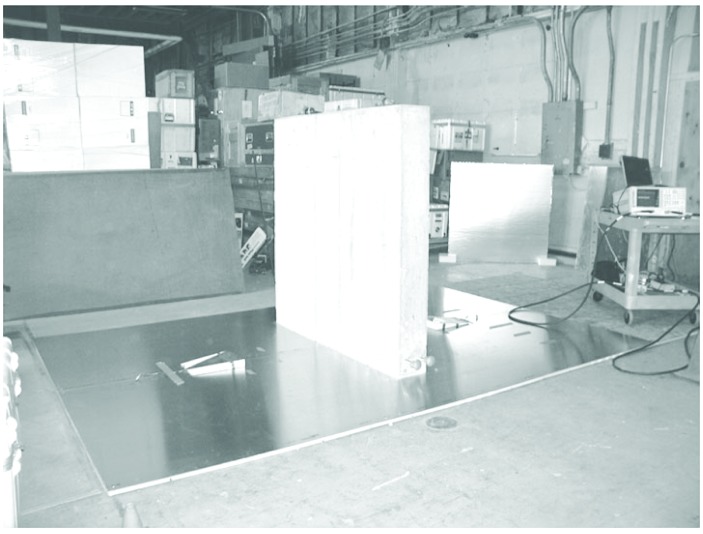
1.2 m × 1.2 m × 0.2 m concrete measurement between TEM half-horns on portable ground-plane.

Figure 1 is a diagram of the laboratory-based measurement system. It consists of an aluminum ground-plane, two TEM half-horn antennas (ground-plane mounted), several material samples, two precision microwave interconnecting cables, and a two-port Vector Network Analyzer (VNA). Four S-parameters (S_11_, S_21_, S_12_, S_22_) are measured at 1601 stepped frequency points in the range of 30 MHz to 6 GHz. For measurements of plywood, gypsum, and cross-linked polystyrene, the 7.3 m × 7.3 m aluminum ground-plane system of the NIST cone and ground-plane time-domain range was used. Measurements of the concrete slab were made on a smaller 3.66 m × 3.66 m aluminum ground-plane. Sample thicknesses were measured with precision calipers at various points around the sample and are accurate to within ±0.1 cm. Figure 2 is a photograph of the measurement system being used on a sample slab of cross-linked polystyrene, and Fig. 3 is a picture of the concrete slab measurement setup. The TEM half-horns can be seen with their styrofoam wedge mounts on either side of the concrete slab.

## 3. Measurement Sequence and Data Processing

The measured S-parameters must be post-processed further before material properties can be extracted. In order to quantify the scattering from the sample under test, three measurements must be made: (1) a background measurement with the two boresighted antennas at a specified separation, (2) a reference reflectivity measurement with a large aluminum sheet halfway between the antennas, and finally, (3) the building material sample placed halfway between the antennas.

The processing consists of three principal steps: (1) The background S-parameters, measured as a function of frequency with no sample in place, are subtracted from the S-parameters measured with either a sample or metal sheet in place; (2) these subtracted S-parameter sets are then inverse Fourier transformed, time gated, and Fourier transformed by NIST-developed software to yield a set of “gated” S-parameters; and (3) the gated S-parameters are then normalized with either the metal reference for reflectivity or with the antenna-to-antenna reference for transmission [2]. Specifically, we have

$$S_{21}=\frac{\left|S_{21m\;\left(gated\;sample\right)}\right|}{\left|S_{21m\;\left(gated\;reference\right)}\right|}$$ (1)

$$S_{11}=\frac{\left|S_{11m\;\left(gated\;sample\right)}-S_{11m\;\left(gated\;reference\right)}\right|}{\left|S_{11m\;\left(gated\;metal\right)}-S_{11m\;\left(gated\;reference\right)}\right|}$$ (2)

$$S_{11}=S_{22};S_{12}=S_{21}.$$ (3)

These three steps yield a set of normalized free-field S-parameters (*S*_11_, *S*_21_, *S*_12_, *S*_22_) that quantify both the reflection and transmission through the sample under test.

## 4. Extraction of Material Electrical Properties

The free-field S-parameters are used in the determinant of the **S**-matrix [3]:

$$|S|=S_{11}S_{22}-S_{21}S_{21}=e^{-2\gamma_{0}\left(d-L\right)}\frac{\Gamma^{2}-z^{2}}{1-\Gamma^{2}z^{2}},$$ (4)

where

$$\gamma_{0}=j\frac{2\pi f}{c_{lab}},$$ (5)

where *f* is the operating frequency,

$$z=e^{-\gamma L}$$ (6)

$$\gamma =j\frac{2\pi f\sqrt{\varepsilon_{r}^{*}}}{c_{vac}},$$ (7)

and

$$\Gamma =\frac{\frac{c_{vac}}{c_{lab}}\sqrt{\frac{\mu_{r}^{*}}{\varepsilon_{r}^{*}}}-1}{\frac{c_{vac}}{c_{lab}}\sqrt{\frac{\mu_{r}^{*}}{\varepsilon_{r}^{*}}}+1}.$$ (8)

In Eqs. (4) through (8), *L* is the measured thickness of the sample, *d* is the antenna separation without a sample present, and *c_vac_* and *c_lab_* [4] represent the speed of light in vacuum and in the laboratory, respectively. Equation (8) contains the complex relative permittivity $\varepsilon_{r}^{*}={\varepsilon^{\prime }}_{r}-j\varepsilon_{r}{}^{\prime \prime }$ and complex relative permeability $\mu_{r}^{*}={\mu^{\prime }}_{r}-j\mu_{r}{}^{\prime \prime }$ of the sample. We assume all sample materials are nonmagnetic and do not have conductive properties, so that ${\mu^{\prime }}_{r}=1$ and ${\mu^{\prime \prime }}_{r}=0$.

Complex relative permittivity is determined by adjusting the value of $\varepsilon_{r}^{*}$ at each frequency to optimize the least-squares difference between the measured results and the theoretical results predicted by Eqs. (4) through (8). The optimizer used for this purpose is a genetic algorithm (GA). In short, the GA [1] is a powerful technique used to minimize the difference between measured and calculated results and, from this, to extract the desired parameters. A GA works by randomly generating many possible solutions. These solutions are tested with the objective function and ranked by a minimum cost criterion. A certain fraction of the best solutions is selected and a new set of solutions is generated. This process is repeated until a desired level of convergence is met or a maximum number of iterations is achieved. A unique property of the GA is that per iteration, a fraction of the solutions are randomly changed to ensure that the results are not subject to local minima. The cost function that is used is the absolute distance per frequency between calculated and measured results and is given by

$$\left||S\left(\varepsilon_{r}{}^{\prime },{\varepsilon^{\prime \prime }}_{r}\right){|}_{f_{i}\left(calc\right)}-|S{|}_{f_{i}\left(meas\right)}\right|.$$ (9)

The first term in Eq. (9) is equivalent to the right-hand side of Eq. (4), and the second term to the left-hand side of Eq. (4). The cost function of Eq. (9) is minimized.

## 5. Results

**Fig. 4:**
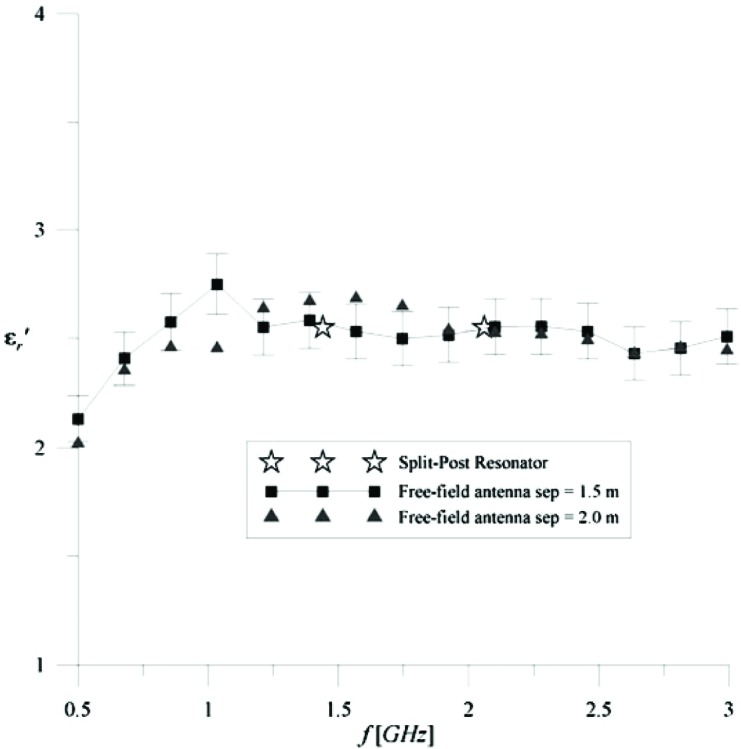
$\varepsilon_{r}{}^{\prime }$ versus frequency for a Split-Post Resonator method [4] and 1.2 m × 1.2 m × 2.9 cm cross-linked polystyrene.

Complex permittivity results are presented for cross-linked polystyrene and concrete. In the case of cross-linked polystyrene, we measured a 1.2 m × 1.2 m sample that was 2.9 cm thick. In this case we know, based on published data obtained by a Split-Post Resonator technique [5], that $\varepsilon_{r}{}^{\prime }=2.55$ at two frequency points, 1.44 GHz and 2.06 GHz. Using this as a guide, the initial population for the genetic algorithm of ${\varepsilon^{\prime }}_{r}$ is drawn from the domain $1<{\varepsilon^{\prime }}_{r}<3$ and the results are shown in Fig. 4. This technique does not have sufficient sensitivity to measure ${\varepsilon^{\prime \prime }}_{r}$ for a low-loss material like cross-linked polystyrene.

**Fig. 5:**
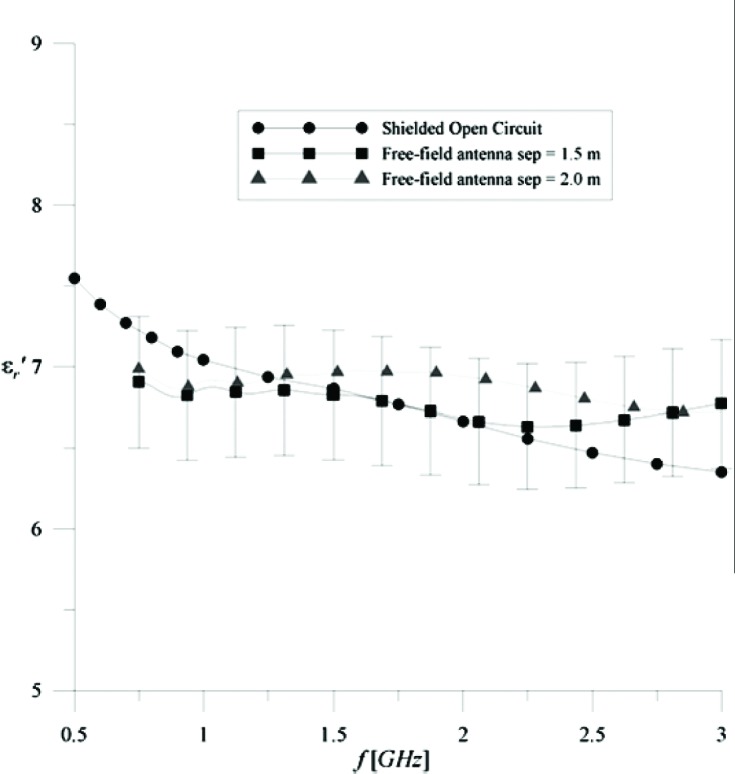
$\varepsilon_{r}{}^{\prime }$ versus frequency for a Shielded Open-circuited method and 1.2 m × 1.2 m × 0.2 m concrete.

**Fig. 6:**
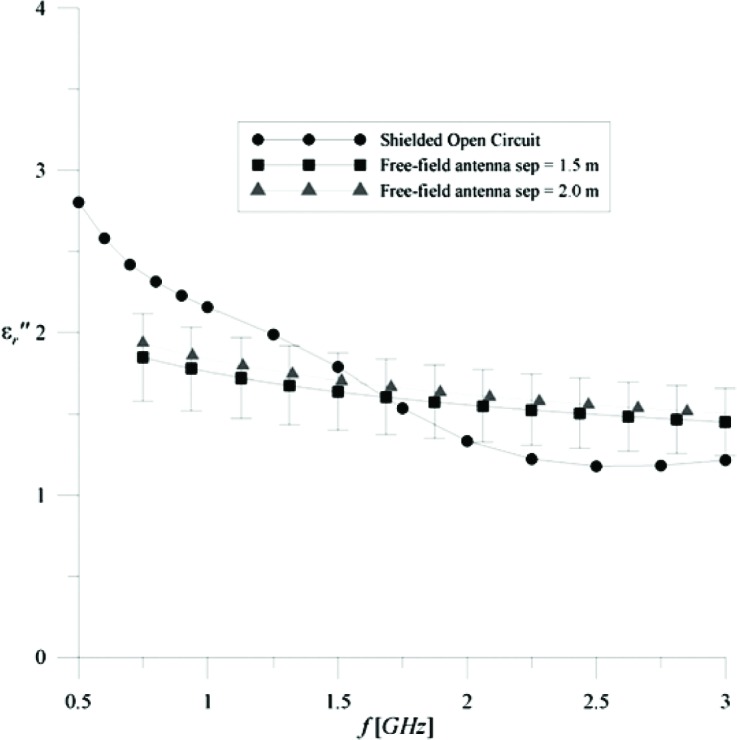
${\varepsilon^{\prime \prime }}_{r}$ versus frequency for a Shielded Open-circuited method and 1.2 m × 1.2 m × 0.2 m concrete.

A series of measurements was also made on a 1.2 m × 1.2 m × 0.2 m section of concrete wall. This sample weighs approximately 907.18 kg and is an ideal candidate for ground-plane based measurements. The results of the concrete measurements are shown in Figs. 5 and 6. The results are in good agreement with independent measurements, using a shielded open-circuit technique conducted by the Electrical Properties of Materials Project, and with published data [2]. The major sources of uncertainty are time-gating errors and possible edge effects that are not gated out of the measurement. Individual source uncertainties are presented in Table 1 where δR and δT are the standard deviations in reflection and transmission signal levels for the stated uncertainties at a frequency of 1 GHz. These individual uncertainties, at each frequency, are RSS combined into reflection and transmission uncertainties, ΔR and ΔT. These uncertainties are propagated into equations for the permittivity uncertainties for reflection and transmission. These permittivity uncertainties are combined in an RSS uncertainty with a coverage factor of 1 (*k* = 1). These uncertainties are shown by the error bars on each plot. All in all, the concrete results are very encouraging. Note also that concrete is highly dispersive and lossy as understood by the imaginary part of permittivity—a result that should be of great interest to the wireless community.

## 6. Conclusions

Utilizing a NIST-developed free-field measurement system, we were able to effectively extract electromagnetic parameters of materials with appropriate signal processing and post-processing using a genetic algorithm. In this process, an objective function chosen to represent the physics of the system is minimized, giving very accurate results. The data obtained should be of great value to the UWB community, and with further research this system promises to be even more accurate, provided that the sources of uncertainty are addressed.

## Figures and Tables

**Table 1:** Sources of uncertainty and their associated values at 1 GHz (*k* = 1)

Source of uncertainty	δR	δT
Repeatability	0.0176	0.0005
Time Gating	0.0187	0.0213
Edge Effects	0.0274	0.0045
Length	0.0049	0.0049
Phase Measurement	0.0042	0.0042

Combined uncertainty (ΔR and ΔT)	0.0381	0.0225
